# Cutaneous granulomatosis and combined immunodeficiency revealing Ataxia-Telangiectasia: a case report

**DOI:** 10.1186/1824-7288-36-29

**Published:** 2010-04-11

**Authors:** Laura Folgori, Alessia Scarselli, Giulia Angelino, Francesca Ferrari, Antonio Antoccia, Luciana Chessa, Andrea Finocchi

**Affiliations:** 1 DPUO, Department of Pediatrics-University of Rome Tor Vergata/Children's Hospital Bambino Gesù, Rome; 2 II School of Medicine, University "Sapienza", Rome; 3 Department of Biology, University Roma Tre, Roma

## Abstract

Ataxia-telangiectasia (A-T) is a complex multisystem disorder characterized by progressive neurological impairment, variable immunodeficiency and oculo-cutaneous telangiectasia. A-T is a member of chromosomal breakage syndromes and it is caused by a mutation in the *ataxia-telangiectasia mutated *(*ATM*) gene. Because of a wide clinical heterogeneity, A-T is often difficult to diagnose in children.

We report an unusual case of a 3-year-old boy affected by A-T who presented exclusively with extensive cutaneous granulomatosis and severe combined immunodeficiency, without neurological abnormalities, at the time of diagnosis. This case clearly emphasizes the variable presentation of A-T syndrome and highlights the difficulties in the early diagnosis of A-T.

A-T should be considered in children with evidence of combined humoral and cellular immunodeficiency associated with unexplained skin granulomatous lesions, even in the absence of the classic features of this syndrome.

## Introduction

Ataxia-telangiectasia (A-T) is an autosomal recessive genomic instability syndrome characterized by progressive cerebellar ataxia, oculo-cutaneous telangiectasia, increased radiosensibility, predisposition to lymphoid malignancies and a variable degree of immunodeficiency. The prevalence is estimated to be between 1:100.000 [1] and 1:40.000 [2]. Both males and females are equally affected.

A-T results from mutations of a single gene, *ATM *(*ataxia-telangiectasia mutated*), located on chromosome 11q22-23 [3,4], encoding a large basic protein involved in cell cycle control and DNA damaging repair.

The diagnosis of A-T is based primarily on clinical findings. Determination of serum alpha-fetoprotein (αFP) is an important diagnostic marker as raised αFP level is found in more than 90% of A-T patients. Confirmatory tests for A-T include colony radiosensitivity assay and identification of the ATM protein by immunoblotting [2,5,6].

We report the case of a 3-year-old boy affected by A-T who presented exclusively with extensive cutaneous granulomatousis and severe combined immunodeficiency, without neurological abnormalities.

## Case Report

A 3-year-old boy was referred to our Department of Pediatrics with a history of cutaneous lesions, recurrent otitis, repeated episodes of fever of unknown origin and suspected immunodeficiency. He was born full term as the second child of healthy non consanguineous parents.

**Figure 1:**
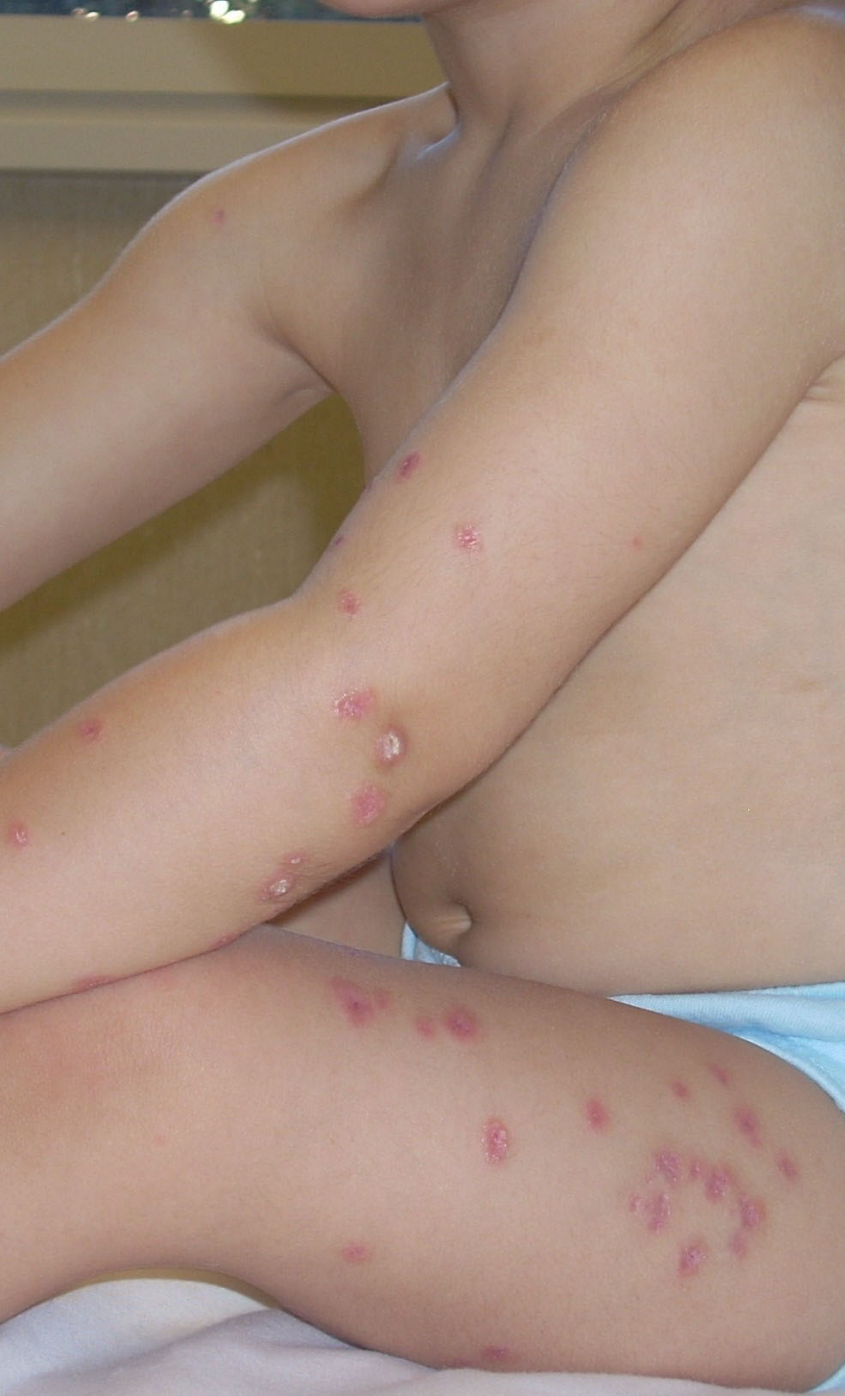
**Cutaneous granulomatous lesions**. Erythematous, scaly dermatitis characterized by small, red and indured lesions on face, arms and legs.

At the age of 2, the child had chickenpox without complications except for a residual erythematous, scaly dermatitis characterized by small, red and indured lesions on face, arms and legs [Fig. 1]. In the suspect of hypersensitivity to insect bites, he was treated with topical treatment (steroids and tacrolimus) and oral antihistamines without improvements.

Our first clinical examination revealed a failure to thrive below the third percentile for height and below the tenth for weight. Chest x-ray showed a lobar pneumonia. Neurological development was normal. Complete blood count (CBC) revealed lymphopenia (range 840-920/ml). Quantitative Polymerase Chain Reaction (PCR) for EBV showed a massive proliferation (1.600.000 copies/ml on blood; 1.000.000 copies/ml on serum) and serological evaluation showed the absence of anti-EBNA IgG. Immunological work-up revealed a combined immunodeficiency [Tab. 1, 2]. Serum IgG and IgA levels were low for the age, as well as IgG2 and IgG4. Specific antibodies response against tetanus toxoid and HiB was low, whereas titer versus Pneumococcal antigens was undetectable despite of three vaccine doses performed. Peripheral blood lymphocyte subsets repeatedly showed a markedly reduced levels of both T and B cells; distribution of naïve and memory T cells showed a marked predominance of T cells with a memory phenotype (CD4+CD45+RO+; CD8+CD45+RO+) and a corresponding reduction of naïve T cells; HLA class I and II expression on lymphocytes was normal. NK cells were very increased (48%) with normal NK activity. Lymphocyte proliferative response to antigens and mitogens was normal, except for a diminished response to PMA+IONO. Thymic output, evaluated by measuring T-cell receptor (TCR) rearrangement excision circles was extremely low in both CD4+ and CD8+ T cells. Analysis of TCR by CDR3 spectratyping revealed oligoclonal expansion in the most of peripheral CD8+ and CD4+ T cells. Skin biopsy showed granulomatous inflammation with an infiltrate characterized by polymorphic lymphocyte and epithelioid cells; Periodic acid-Schiff (PAS), Giemsa, and Ziehl-Neelsen staining of skin-biopsy specimens showed no evidence of fungi or mycobacteria and PCR assays for mycobacterium tuberculosis were negative. *In situ *analysis of TCR repertoire of CD3 lymphocyte resulted completely skewed.

**Table 1:** Immunological data

Parameter	Results	Age matched controls
WBC/ml	5.700	5-16 × 10 ^3^
Hb gr/dl	12	11.5-13
Plts/ml	370.000	250-550 × 10 ^3^
		
Serum immunoglobulin mg/dl		
IgA	10	27-173
IgM	61	62-257
IgG	293	462-1710
IgG1	287	280-830
IgG2	9	40-240
IgG3	2.4	6-130
IgG4	0.3	3-120
		
Lymphocyte phenotype		
Absolute count/ml	840	1.7-6.9 × 10 ^3^
CD3	304 (36.3%)	0.9-4.5 × 10 ^3^
CD4	121 (14%)	0.5-2.4 × 10 ^3^
CD4RA	22%	
CD4RO	78%	
CD8	153 (18.3%)	0.3-1.6 × 10 ^3^
CD8RA	29%	
CD8RO	71%	
CD19	42 (4.9%)	0.2-2.1 × 10 ^3^
CD16-56	467 (55.7%)	0.1-1 × 10 ^3^
TRECs		
CD4	absent	1.106 - 15.787
CD8	absent	1.106 - 15.787
		
Response to mitogens and antigens (counts per minute)		
none	439	> 430
PHA	33775	> 35000
OKT3	34410	>25000
PMA+IONO	4772	>25000
none	3069	>1250
Candida	9711	>5000
Tetanus toxoid	3231	> 5000

**Table 2:** Serological data after 3 vaccine doses

Parameter	Results	Reading
Tetanus toxoid IU/ml	0.1	>0.1 IU/ml
Haemophilus influenzae B mg/l	0.1	>1 μg/ml
S. Pneumoniae mg/l	3	>20 mg/l

Intravenous immunoglobulin infusion at the dosage of 400 mg/Kg monthly and prophylactic treatment with trimethoprim-sulfamethoxazole was started; during this period a marked reduction of EBV viral load was observed. Six months later the child experienced a bilateral acute otitis that required a long and multiple antibiotic therapy.

Based on the male-gender, hypogammaglobulinemia and high EBV replication rate, we firstly ruled out Lymphoproliferative X-linked Syndrome (XLP) and XIAP deficiency by molecular analysis [7]. Schuetz et al. reported a hypomorphic heterozygous RAG mutation with skin granulomatous lesions associated with secondary complications of EBV infection and severe immunological abnormalities [8]; molecular analysis for RAG1/2 resulted negative. Even in the absence of a severe clinical picture, immunological B and T cells alterations led us to consider a leaky SCID. Sequencing of common γ-chain and IL7-R genes and dosage of ADA enzyme in white blood cells and plasma resulted normal. There was no evidence of maternal engraftment in the patient's peripheral blood by chimerism analysis.

In the suspect of a double strands break repair defect, radiosensitivity test was performed, showing an increased number of chromosomal aberrations. Alpha-fetoprotein (αFP) serum value repeatedly resulted increased (135 ng/ml, n.v. <10 ng/ml) and Western Blotting showed the absence of ATM protein.

Characterization of the ATM gene mutations are in progress.

Phenotypic heterogeneity has long been identified in both laboratory and clinical features of A-T, ranging from the classic form with typical symptoms to unusual disease presentation. Neurological involvement (i.e. cerebellar ataxia and dysarthria) and ocular telangectasia are the hallmarks of A-T. Nevertheless, milder forms of A-T, which are characterized by late onset or slow progression of neuromotor dysfunction, have been described and diagnosed even among adults during evaluation on unknown ataxia. The typical cutaneous manifestation of the disease is ocular telangectasia although some patients have no telangectasia, even in adulthood; cutaneous granulomatosis with no identifiable infectious origin occurs rarely in children with primary immunodeficiency [8-10]. These cutaneous granulomas have been previously described in common variable immunodeficiency [9,11-13], chronic granulomatous disease, X-linked hypogammaglobulinemia [14,15] but rarely in A-T [16-19]. Furthermore, we did not see resolution of granulomas after neither the local immunosuppressive therapy, nor the treatment with intravenous immunoglobulin, as it has been reported in some patients with common variable immunodeficiency [9,20,21].

Similarly to the phenotypic features, a wide variability of immunological defects exists in patients with A-T syndrome, ranging from the absence of immune defects to severe immunodeficiency with recurrent infections [22,23]. Low levels of IgG, IgA and IgE are observed in more than 80% of the patients, whereas severe combined immunodeficiency with recurrent infections occur rarely. In our patient, immunological abnormalities were so severe that led us to suspect a combined immunodeficiency. Diagnosis led us to reveal A-T even in child's elder sister (8-years-old) for whom a wrong spastic paresis diagnosis was previously made elsewhere. She conversely presented a common clinical phenotype of A-T, showing progressive neurological degeneration with ataxia, dysarthria, dyskinesia, dystonia and oculo-cutaneous telangiectasia. Her immunological work-up only revealed a mild lymphopenia with an IgA defect. The different clinical picture seen in patients with defects of that same gene can be caused by specific mutations, other genetic factors or epigenetic mechanism. Previous studies have failed to indicate a specific correlation between the amount of ATM protein and radiosensitivity. However, the results of various studies suggest a clear correlation between preservation of neurological function, decreased radiosensitivity and level of normal ATM protein kinase activity. Interestingly, there are siblings, as our patients, who reported absent ATM protein but clinically and laboratory distinct phenotypes [23,24]. Thus, our case supports the lack of a strict genotype-phenotype correlation[2,22-26] suggesting that the heterogeneity of A-T is much broader than initially recognized and that A-T represents a complex spectrum of the disease shaped by the genetic mutations, host characteristics, pathogens (EBV) and environmental factors.

The prevalence of the disease reported in the literature is very variable and estimated to be 1:40,000-100,000 births [1,2]; this is probably due to the wide clinical heterogeneity that often leads physicians to an incorrect or missed diagnosis. Atypical presentation of our patient caused a delay of diagnosis; there should be a high index of suspect for A-T among children with recurrent infections and immunodeficiency, even in the absence of neurological abnormalities. Accordingly, screening with serum alpha-fetoprotein, a simple, rapid and reliable test for A-T, should be part of the evaluation of children with any congenital immunodeficiency. A delayed diagnosis can often affect the clinical evolution and influence the management of A-T patients and their relatives [1,27]. Early diagnosis alerts the physicians to limit ionizing radiations exposure, including the diagnostic methods. Also, early diagnosis allows genetic counseling as well as the identification of the carrier parents who may have an increased cancer risk, particularly breast cancer in women [1,28].

## Conclusion

In conclusion, A-T syndrome should be considered in children with an evidence of combined humoral and cellular immunodeficiency and otherwise unexplained granulomatous skin lesions.

## Competing interests

The authors declare that they have no competing interests.

## Authors' contributions

LF and AS were involved in clinical care of patient and contributed to the writing of the paper. GA were involved in clinical care and in the collection of clinical data of the patient. LC and FF performed Western Blot analysis. AA performed the Radiosensitivity Test. AF has defined the clinical picture of the patient, formulated diagnosis and wrote the paper. All authors have read and approved the final manuscript.

## Consent

Written informed consent was obtained from the patient's relatives for publication of this case report.
